# Metachronous solitary splenic metastasis arising from early gastric cancer: a case report and literature review

**DOI:** 10.1186/s12893-017-0292-0

**Published:** 2017-08-29

**Authors:** Tsutomu Namikawa, Yasuhiro Kawanishi, Kazune Fujisawa, Eri Munekage, Masaya Munekage, Takahito Sugase, Hiromichi Maeda, Hiroyuki Kitagawa, Tatsuya Kumon, Makoto Hiroi, Michiya Kobayashi, Kazuhiro Hanazaki

**Affiliations:** 1 Department of Surgery, Kochi Medical School, Kohasu, Oko-cho, Nankoku, Kochi 783-8505 Japan; 2 Department of Pathology, Kochi Medical School, Kochi, Japan; 3 Cancer Treatment Center, Kochi Medical School Hospital, Kochi, Japan; 4 Department of Surgery, Noichi Central Hospital, Kochi, Japan; 5 Department of Human Health and Medical Sciences, Kochi Medical School, Kochi, Japan

**Keywords:** Gastric cancer, Splenic metastasis, Gastrectomy, Solitary metastasis, Splenectomy

## Abstract

**Background:**

The metastasis of malignant tumors to the spleen is rare, and only a small percentage of cases can be treated surgically, as splenic metastases generally occur in the context of multivisceral metastatic cancer at a terminal stage. We report a rare case of metachronous solitary splenic metastasis arising from early gastric cancer.

**Case presentation:**

A 75-year-old man was initially referred to our hospital for examination of gastric cancer, diagnosed at a medical check-up. Esophagogastroduodenoscopy showed a slightly elevated lesion with a central irregular depression in the upper-third of the stomach. Biopsy specimens of the lesion showed a moderately-differentiated adenocarcinoma, and abdominal computed tomography showed no evidence of distant metastases. Endoscopic submucosal dissection was performed, with histological confirmation of a moderately-differentiated adenocarcinoma invading the submucosal layer. The patient subsequently underwent laparoscopic total gastrectomy with regional lymph node dissection, resulting in no residual carcinoma and no lymph node metastasis. Computed tomography, 28 months later, showed a well-defined mass measuring 4.2 cm in diameter in the spleen, and the patient underwent a splenectomy, since there was no evidence of further metastatic lesions in any other organs. Histological examination confirmed the diagnosis of a poorly-differentiated adenocarcinoma originating from the previous gastric cancer. The patient was alive 2 months after surgical resection of the splenic metastasis without any recurrence.

**Conclusion:**

To the best of our knowledge, this is only the second case of a solitary splenic metastasis from early gastric cancer to be reported in the English literature. The present case suggests surgical resection may be the preferred treatment of choice for patients with a solitary splenic metastasis from gastric cancer.

## Background

The splenic metastasis arising from other malignancies is rare disease, which surgical treatment is often difficult due to presentation as multivisceral metastatic status in an advance progressed stage [1]. Previous literature demonstrated that the incidence of splenic metastasis from other solid malignancies including sarcoma or carcinoma was only 1.3% in a series of 1280 splenectomies [2]. Although splenic metastasis may originate from various organs, it has been reported that the most common primary organs of splenic metastases are breast, lung, colorectal, and ovarian carcinomas [3]. On the other hand, early gastric cancer (EGC) defined as a lesion confined to the mucosa or submucosa, independent of the lymph node metastasis, has favorable outcomes without hematogenous recurrence by the optimal surgical treatment [4].

In this report, we describe the case of a 77-year-old man who presented 28 months after gastrectomy for EGC, with a solitary splenic metastasis which was successfully treated by a splenectomy. The clinical characteristics of previously reported cases are also discussed.

## Case presentation

**Fig. 1:**
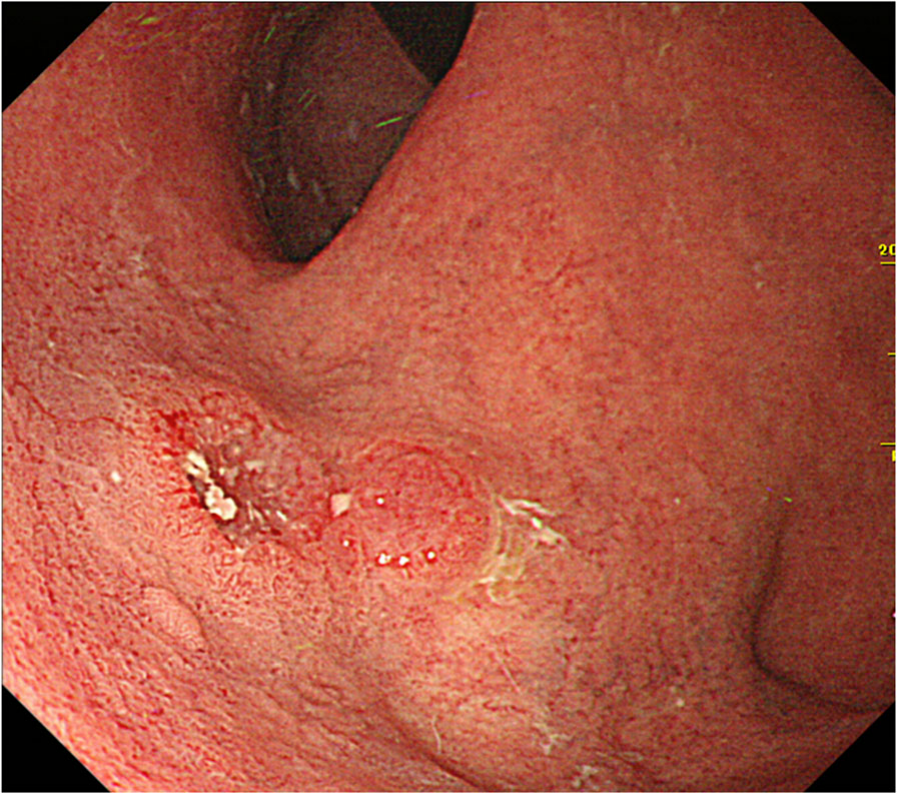
Esophagogastroduodenoscopy showing a slightly elevated lesion with central depressed area in the upper-third of the stomach

A 75-year-old Japanese man was referred to our hospital for further examination of gastric cancer diagnosed at a medical check-up. His past medical history revealed that he had undergone endoscopic submucosal dissection (ESD) 2 years earlier for EGC confined to the mucosa. On admission, his laboratory results, as well as serum carcinoembryonic antigen and cancer antigen 19–9 were almost within normal limits. Esophagogastroduodenoscopy revealed an elevated lesion with a central irregular depression in the upper-third of the stomach, measuring 2.2 cm, which proved to be a well-differentiated adenocarcinoma on biopsy (Fig. 1).

**Fig. 2:**
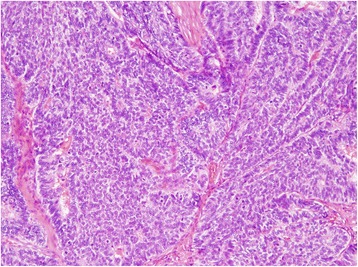
Histological examination of the resected specimen by endoscopic submucosal dissection showing a well-differentiated adenocarcinoma coexisting with a solid-type poorly-differentiated adenocarcinoma. Stained with hematoxylin and eosin

Abdominal computed tomography (CT) showed no evidence of distant metastases and we performed ESD under a clinical diagnosis of EGC. The histological findings showed a well-differentiated adenocarcinoma coexisting with a solid-type poorly-differentiated adenocarcinoma, invading the submucosal layer more than 2 mm (Fig. 2). Therefore, in accordance with Japanese gastric cancer treatment guidelines [5], the patient subsequently underwent laparoscopic total gastrectomy with regional lymph node dissection, resulting in no residual carcinoma, lymph node metastasis, or lymphovenous invasion. The postoperative course was uneventful, and he was discharged on postoperative day 14.

**Fig. 3:**
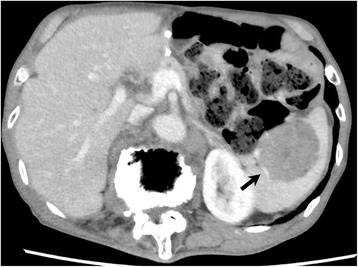
Abdominal computed tomography showing a well-defined mass in the spleen measuring 4 cm in diameter (*arrow*)

**Fig. 4:**
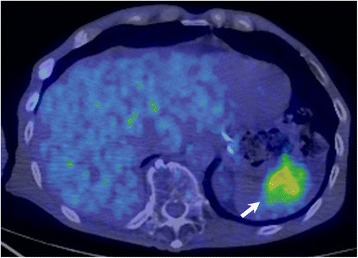
^18^F-2-deoxy-2-fluoro-glucose (FDG) positron emission tomography combined with computed tomography imaging showing the splenic mass with intense FDG uptake *(arrow)*

**Fig. 5:**
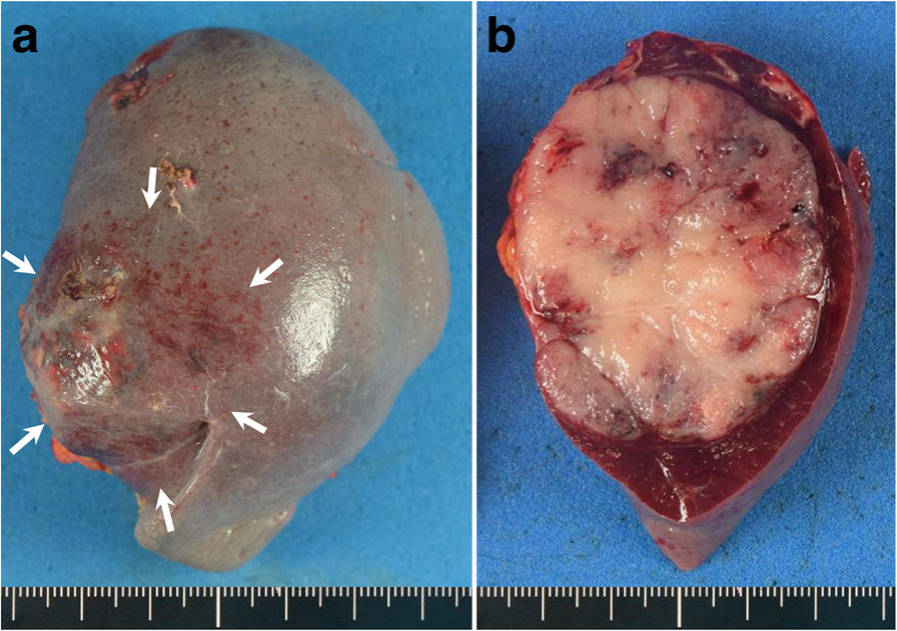
Gross examination of the surgically resected specimen showing a discolored surface to the spleen caused by the tumor (**a**, *arrows*), which is a well-circumscribed solid tumor measuring 4.2 cm (**b**)

The patient underwent periodic follow-up physical examinations, which were blood tests and CT performed every 6 months. At 28 months after the operation, abdominal CT revealed a well-defined mass measuring 4.2 cm in diameter in the spleen (Fig. 3, *arrow*). ^18^F-2-deoxy-2-fluoro-glucose (FDG) positron emission tomography combined with CT imaging showed intense FDG uptake in the splenic mass, with a maximum standardized uptake value of 6.1 (Fig. 4, *arrow*). As there was no evidence of further metastatic lesions in any other organs, a splenectomy was performed. The gross appearance of the surgically-resected specimen showed a discolored surface to the spleen with a slightly irregular surface caused by the tumor (Fig. 5a, *arrow*). On cross-section, the specimen showed a well-circumscribed, solid tumor measuring 5.5 × 4.5 cm in diameter (Fig. 5b).

**Fig. 6:**
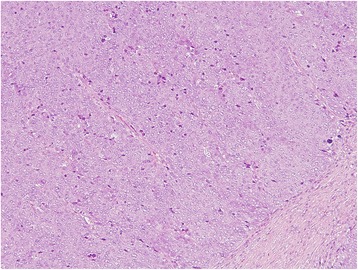
Histological examination of the resected specimen demonstrating a solid-type poorly-differentiated adenocarcinoma originating from the previous gastric cancer. Stained with hematoxylin and eosin

Histological examination confirmed the diagnosis of a solid-type poorly-differentiated adenocarcinoma, consistent with the features of the primary gastric cancer. The tumor was in the splenic parenchyma and was totally covered with splenic peritoneum (Fig. 6). The results of immunohistochemical investigations of both the primary early gastric cancer and the splenic tumor showed negative immunostaining for chromogranin A, synaptophysin, MUC1, MUC2, MUC5AC, and MUC6. No tumor cells were detected in the lymph nodes of the splenic hilum, and there was no reactivity for human epidermal growth factor receptor 2 in either the primary gastric cancer or splenic metastasis. The postoperative course was uneventful, and the patient has been well without evidence of recurrence for 2 months following the splenectomy.

## Discussion and conclusions

This report describes the rare case of a patient with a solitary splenic metastasis arising from EGC, treated by splenectomy. A search of English language publications between 2000 and 2016 was conducted using the Medline and PubMed databases for articles on splenic metastases from gastric cancer, with the keywords “gastric cancer” and “splenic metastasis”. Data on age, gender, tumor location, tumor size, depth of invasion, histological type, treatment, and outcome for each reported case were obtained. To the best of our knowledge, this is only the fourth case of a solitary splenic metastasis arising from gastric cancer, and the second case arising from EGC to be reported in the English literature.

The clinicopathological features of the three previously reported cases [6–8] and the present case are listed in Table 1. The median age of these four patients was 70 years (range, 49–75 years), and all patients were male. Gastric cancer was reported in the upper-third of the stomach in two patients (both EGC), while one patient had a lesion in the middle-third of the stomach, and one had a lesion in the lower-third of the stomach (both advanced gastric cancer).

**Table 1:** Clinicopathological data from reported cases of solitary splenic metastasis arising from gastric cancer

Author	Age	Gender	Primary gastric cancer							Splenic metastasis				Outcome
			Tumor location	Tumor size (cm)	Gross appearance type	Tumor depth	Lymph node involvement	Histological type	Treatment	Tumor size (cm)	Treatment	Histological type	Duration (months)	
Yamanouchi [6]	65	Male	L	ND	Ulcerated	ss	Positive	Intestinal	DG	4.5	Splenectomy	Intestinal	50	DOD at 40 months
Kawasaki [7]	75	Male	U	2.0	Elevated	sm	Positive	Intestinal	EMR, PG	6.5	Chemotherapy, Splenectomy	Intestinal	14	2 years survival
Deng [8]	49	Male	M	6.0	ND	se	Positive	Intestinal	DG	14	Splenectomy	Diffuse	60	9 months survival
Present case	77	Male	U	2.2	Elevated	sm	Negative	Intestinal	ESD, TG	5.5	Splenectomy	Diffuse	28	2 months survival

*Abbreviations*: *DG* distal gastrectomy, *DOD* dead of disease, *EMR* endoscopic mucosal resection, *ESD* endoscopic submucosal dissection, *L* lower-third of the stomach, *M* middle-third of the stomach, *ND* not described, *PG* proximal gastrectomy, *se* serosa, *sm* submucosa, *ss* subserosa, *TG* total gastrectomy, *U* upper-third of the stomach

Treatment consisted of total gastrectomy in one patient, distal gastrectomy in two patients, and proximal gastrectomy in one patient. The two patients with EGC were initially treated endoscopically, such as by endoscopic mucosal resection or ESD. The median tumor size of the primary gastric cancer was 2.2 cm (range, 2.0–6.0 cm), and histological analysis of the gastric tumors revealed intestinal-type carcinomas in all four cases. Regional lymph node metastasis was detected in both cases of advanced gastric cancer and in one case of EGC. The median duration between the initial gastrectomy and the appearance of the splenic metastasis was 39 months (range, 14–60 months). All the patients were treated by surgical resection for the splenic metastasis, and the median tumor size of the splenic metastasis was 6.0 cm (range, 4.5–14 cm). One patient who was initially diagnosed with advanced gastric cancer died from multiple metastases to the liver and peritoneal dissemination 40 months later.

Both the patients with a solitary splenic metastasis arising from EGC were initially treated by endoscopic resection, but subsequently underwent radical gastrectomy with regional lymphadenectomy due to submucosal invasion of the tumor. Lymph node metastasis may contribute to the occurrence of splenic metastases, because it is reported to be the most important risk factor to occur the recurrence of EGC [4, 9, 10]. However, there was no lymph node metastasis or lymphovascular infiltration in our present case, while the previously reported case of EGC showed only one lymph node metastasis. It remains uncertain whether there is any association between initial endoscopic treatment for EGC and splenic metastases. Although a solitary splenic metastasis from gastric cancer is extremely rare, either synchronous or metachronous to the primary tumor, surgical resection might be the effective strategy to treat patients with a solitary splenic metastasis from EGC.

It is a quite difficult issue to diagnose whether the splenic mass lesion is a primary tumor or a metastatic tumor. The differential diagnosis of a splenic mass without the context of active cancerous disease would include a primary splenic lesion, such as lymphoma, hemangioma or lymphangioma. The clinical diagnosis of splenic metastases seems to be largely dependent on the previous history of malignant disease in the patients [8]. Therefore, a splenectomy in the case of a splenic metastasis makes sense, if the metastasis is isolated [1]. Although the reason for the rarity of splenic metastases arising from solid malignant tumors remains uncertain, even though the spleen is a hypervascular organ, the growth of an early blood-borne micrometastasis may contribute its occurrence [3].

A solitary splenic metastasis arising from EGC is extremely rare, as metastases to the spleen are usually found in conjunction with metastases to other organs. However, even in cases of EGC, a solitary metastasis to the spleen should not be discounted during the periodic follow-up examinations of patients with gastric cancer. When treating metastatic or recurrent gastric cancer, clinicians should always consider the adverse clinical effects and stress of surgical resection, however, a splenectomy might be a potentially effective treatment in the case of a solitary metastasis. Further studies and the assessment of additional cases are needed to establish standardized recommendations for the management of this rare entity.

## Abbreviations

CT: Computed tomography
EGC: Early gastric cancer
ESD: Endoscopic submucosal dissection
